# Tracing environmental contaminants in magellanic penguins: Legacy POPs and metabolic clearance of PAHs

**DOI:** 10.1007/s11356-026-37785-x

**Published:** 2026-04-30

**Authors:** Thales Henrique de Carvalho Storti, Antonio Derley de Sousa Pereira, Cristian Taboada Timoszczuk, Raphael de Lucca Marcello Jarcovis, Lígia Dias de Araujo, Daniela Alves Maia da Silva, Felipe Rodrigues dos Santos, Josilene da Silva, Satie Taniguchi, Rafael Andre Lourenço

**Affiliations:** 1 University of Sao Paulo Institute of Oceanography: Universidade de Sao Paulo Instituto Oceanografico. São Paulo, São Paulo, Brazil; 2 Federal University of Rio Grande Oceanography Institute: Universidade Federal Do Rio Grande Instituto de Oceanografia. Rio Grande, Rio Grande Do Sul, Brazil

**Keywords:** Persistent organic pollutants, Magellanic penguins, Organochlorine pesticides, Marine contamination, Environmental monitoring, Seabirds

## Abstract

Persistent organic pollutants (POPs) are widespread contaminants that bioaccumulate in the food web, posing risks to wildlife, especially top predators. This study investigates the occurrence of POPs in liver tissues of 23 Magellanic Penguins (*Spheniscus magellanicus*) stranded along the Brazilian coast from June to September 2017. We analyzed concentrations of polychlorinated biphenyls (PCBs), organochlorine pesticides (DDTs, Drins, mirex, endosulfans), and hexachlorobenzene (HCB). POPs were detected in all samples. PCBs and DDTs showed the highest concentrations, with PCBs ranging from < LOD to 199.4 ng g^−1^ ww (mean: 57.2 ± 49.0 ng g^−1^ ww), showing a declining trend compared to previous reports. DDT metabolites, especially p,p′-DDE, remained persistent in the samples. HCB was found in 70% of the individuals but at lower levels than previously reported. Other pesticides were present at trace levels, consistent with their historical use in Brazil. No polycyclic aromatic hydrocarbons (PAHs) were detected, which we attribute to rapid metabolic clearance in penguins rather than lack of exposure. This emphasizes the species’ efficiency in eliminating these non-persistent compounds. Our findings highlight the continued presence of legacy POPs in the marine ecosystem and their bioaccumulation in seabirds. Although PCB levels appear to be decreasing, these pollutants still pose ecological risks. Monitoring efforts remain essential, especially considering the potential for re-exposure due to global distillation and melting of polar ice. This study contributes to understanding contaminant dynamics in the Southern Hemisphere and underscores the importance of long-term monitoring of marine wildlife.

**Supplementary Information:**

The online version contains supplementary material available at 10.1007/s11356-026-37785-x.

## Introduction

The presence of POPs in the environment may be one of the factors affecting aquatic animals’ health. POPs include synthetic substances from various chemical groups that have been deliberately or inadvertently introduced into the environment. Due to their stability, long-range transport potential, and toxicity, they are of significant environmental concern (Xu et al. 2013). POPs pose risks to both human health and the environment, as they remain unaltered for exceptionally long periods, become widely distributed through natural processes involving soil, water, and air, accumulate in the fatty tissues of living organisms (including seabirds), biomagnify at higher trophic levels, and are toxic to various organisms, as recognized by the Stockholm Convention (UNEP 2019).

In parallel, PAHs represent another major class of contaminants of concern in marine ecosystems. These compounds originate mainly from pyrogenic and petrogenic sources and are widely distributed in the environment (Balmer et al. 2019). PAHs tend to associate with suspended particles and sediments, from where they can enter aquatic food webs. Although vertebrates, including penguins, generally possess enzymatic pathways that enable the biotransformation and excretion of PAHs, reducing their potential for biomagnification, these compounds are still relevant due to their persistence, bioavailability, and capacity to induce toxic, mutagenic, and carcinogenic effects (Neff 1979; Law and Whinnett 1992; Jonsson et al. 2004).

As top predators in the marine food chain, seabirds, including penguins, are widely used as bioindicators to study the occurrence and distribution of environmental pollution (Furness and Camphuysen 1997; Burger and Gochfeld 2004; Zhang et al. 2007; Ellis et al. 2018; Morales et al. 2022; Pala et al. 2024, 2025; Motas et al. 2025a, 2025b). Penguins are oceanic birds of the order Sphenisciformes, characterized by their inability to fly due to the evolution of their wings into flippers and the absence of pneumatic bones. Adapted to aquatic life, they use their wings for propulsion and can remain submerged for several minutes. Additionally, their vision is specialized for diving, enhancing their efficiency as skilled hunters (Sick 1997; Orr 2000; Pough et al. 2003).

Currently, between sixteen and nineteen penguin species are known, all of which are found in the Southern Hemisphere. Along the Brazilian coast, *Spheniscus magellanicus* (Forster, 1781), commonly known as the Magellanic Penguin, is frequently observed, predominantly as juveniles (Sick 1997; Vooren and Brusque 1999; Orr 2000; Rezende et al. 2013). *Spheniscus* penguins have a reproductive season from November to January, during which they spend most of their time in Argentine and Chilean waters, only venturing into the sea for short feeding periods (Frere et al. 1996; Gandini et al. 1999). This is followed by an oceanic phase, primarily from March to September, when they migrate north and winter on the continental shelf off the coasts of Uruguay and Brazil (Stokes et al. 1998; Pazos et al. 2003; Pütz et al. 2007). At the reproduction time, females lay two eggs between October and November, with most hatching between mid-November and early December.

Their diet consists mainly of fish, with some consumption of cephalopods and crustaceans. Their feeding behavior changes between the reproductive and pelagic periods, shifting from stenophagic (a restricted diet) to euryphagic (a varied diet with no preference for type or size), increasing dietary diversity. This shift is particularly evident when juveniles reach Brazilian waters. Post-mortem examinations conducted by the non-governmental organization SOCOBIOMA since 2006 have found no cephalopod beaks in their stomach contents, an unusual finding, given the common presence of cephalopods in both the diet and the frequency of stranded animals in Brazil (Pinto et al. 2007; Baldassin et al. 2010).

During their months at sea searching for food, Magellanic penguins rely on fat reserves as an energy source (Warham 1990). Consequently, accumulated chemical contaminants are mobilized into other tissues (Tanabe 1988), potentially leading to rapid liver contamination (Elliott et al. 1996). Archaeological studies of shell middens along the Brazilian coast have revealed layers of shells, arrowhead fragments, axes, ceramics, human skeletons, and animal bones, including penguin remains, indicating that these birds reached the Brazilian coast long before Portuguese colonization (Gaspar 1999).

Specifically, persistent organic pollutants (POPs) and polycyclic aromatic hydrocarbons (PAHs) have been found in Magellanic penguins stranded in Brazilian coast and other regions (Baldassin et al. 2016; Barreto et al. 2020; Quinete et al. 2020; Fernandez et al. 2025). In this context, the present study seeks to evaluate POPs and PAHs in liver tissue extracted from *Spheniscus magellanicus* carcasses found along the coast of Santa Catarina, Brazil, thereby contributing to a better understanding of the dynamics of these pollutants in the Southern Hemisphere and their potential impact on wildlife.

## Materials and methods

### Sampling

**Fig. 1:**
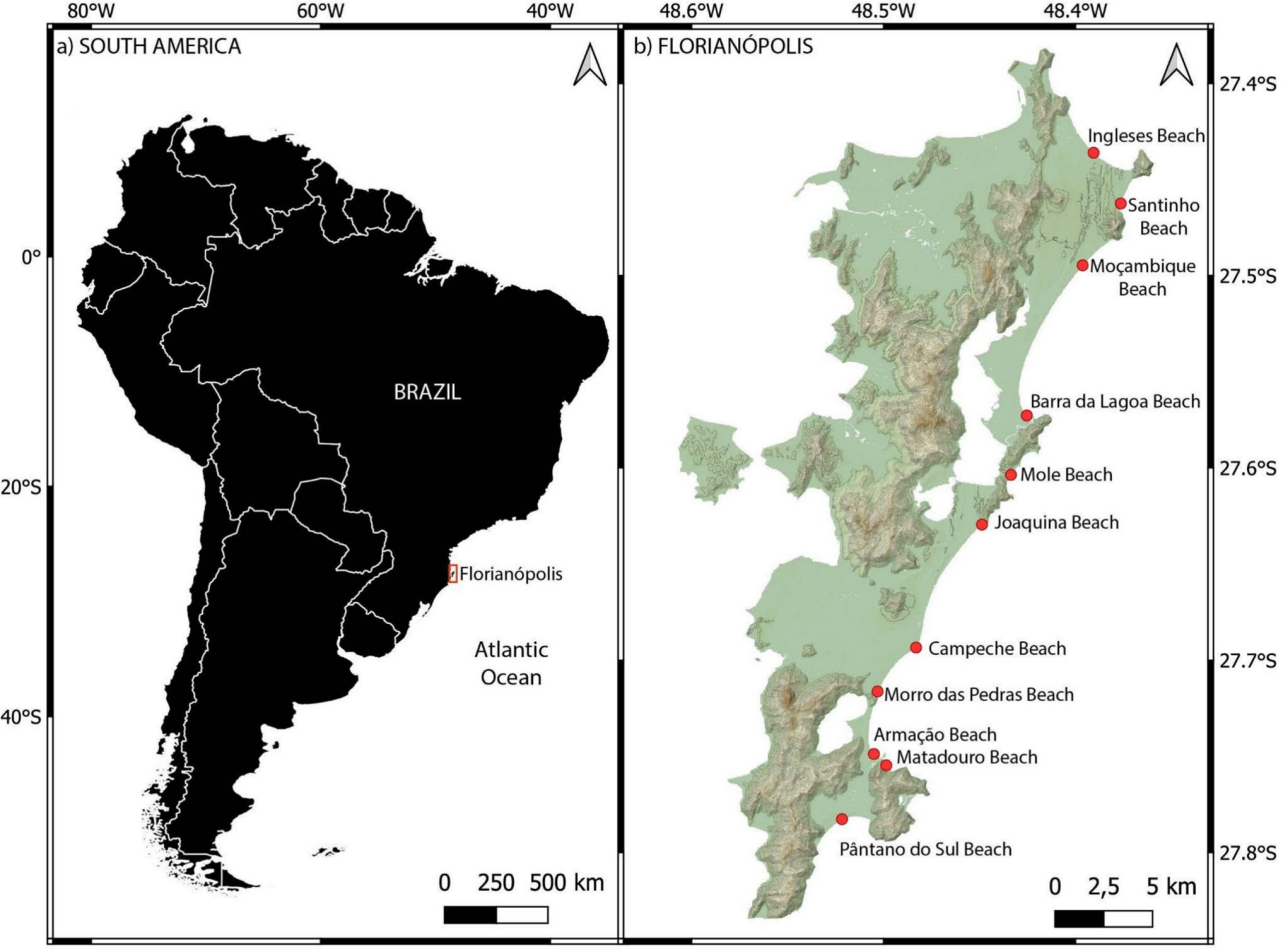
Sites of collect of *Spheniscus magellanicus* carcasses in the island of Florianópolis, Brazil

During the austral winter, from June to September 2017, 23 *Spheniscus magellanicus* juveniles’ carcasses were collected from the beaches of Florianópolis, a coastal city located in an island in southern Brazil (Fig. 1). These consisted of 6 males and 17 females, all carcasses in good condition (code 2 according to Geraci and Lounsbury (1993)) and exhibiting thinness or cachexia, with body weights ranging from 1.6 to 2.5 kg. Necropsies and tissue collection, including liver samples, were performed at veterinary centers. Tissue collection was authorized by IBAMA (Brazilian Institute of Environment and Renewable Natural Resources) under licenses Abio No. 640/2015 and Abio No. 755/2016. All samples were stored in an ultra-freezer at −80 °C until analysis.

The analyzed POPs included polychlorinated biphenyls (PCBs), organochlorine pesticides, and polybrominated diphenyl ethers (PBDEs). The PCB congeners examined were PCB 49, PCB 52, PCB 66, PCB 77, PCB 81, PCB 95, PCB 101, PCB 110, PCB 114, PCB 118, PCB 123, PCB 138, PCB 141, PCB 149, PCB 151, PCB 153, PCB 156, PCB 157, PCB 169, PCB 174, PCB 177, PCB 180, PCB 189, PCB 194, PCB 195, and PCB 206. The analyzed organochlorine pesticides included hexachlorobenzene (HCB), hexachlorocyclohexanes (α-, β-, δ-, and γ-HCH), DRINs (aldrin, isodrin, dieldrin, and endrin), chlordanes (heptachlor, heptachlor epoxide A, heptachlor epoxide B, oxychlordane, α- and γ-chlordane), dichlorodiphenyls (o,p'-DDE, p,p'-DDE, o,p'-DDD, p,p'-DDD, o,p'-DDT, p,p'-DDT), endosulfans I and II, methoxychlor, and mirex. For organobromine compounds, the PBDE congeners analyzed were PBDE 28, PBDE 47, PBDE 99, PBDE 100, PBDE 153, PBDE 154, and PBDE 183. Furthermore, in addition to POPs, 38 parent PAHs and their alkylated substituted groups were analyzed, which included the 16 EPA priority PAHs (naphthalene, acenaphthylene, acenaphthene, fluorene, phenanthrene, anthracene, fluoranthene, pyrene, benz[*a*]anthracene, chrysene, benzo[*b*]fluoranthene, benzo[*k*]fluoranthene, benzo[*a*]pyrene, benzo[*a*]pyrene, indeno[1,2,3-*cd*]pyrene, dibenz[*a*,*h*]anthracene, benzo[*g*,*h*,*i*]perylene, dibenzothiophene, perylene) and their alkylated counterparts (C1 to C4-naphthalenes, C1 to C3-fluorenes, C1 to C3-dibenzothiophenes, C1 to C4-phenanthrenes-anthracenes, C1 to C2-fluoranthene-pyrenes and C1 to C2-chrysenes).

Analytical procedures followed the guidelines of the U.S. Environmental Protection Agency (USEPA), with adaptations based on the National Oceanic and Atmospheric Administration (NOAA) protocol described by Wade and Cantillo (1996) and more recent literature (Timoszczuk et al. 2025). The analytical procedure is described in detail in the Supplementary material. Briefly, the analytical process began with moisture content determination via gravimetry. Lipid extraction was performed on a separate sub-sample using the USEPA 3540 C method, involving Soxhlet extraction with n-hexane and dichloromethane. Lipid content was quantified gravimetrically following solvent evaporation, in accordance with USEPA 8290 standards. Extract purification followed a combination of USEPA methods (3610, 3630, 3640), utilizing silica gel and alumina columns, with additional refinement via high-performance liquid chromatography (HPLC). PCBs, PBDEs and PAHs were quantified using gas chromatography-mass spectrometry (GC–MS) under USEPA 8270E guidelines, while organochlorine pesticides were analyzed via gas chromatography with electron capture detection (GC-ECD) following USEPA 8081B. Internal standardization and calibration curves were applied, with detection and quantification limits established based on standard deviations and calibration points.

To ensure the reliability of the results, a quality assurance and quality control (QA/QC) procedure, based on Wade and Cantillo (1996), was implemented, including the analysis of method blanks, fortified blanks, fortified matrix samples, sample duplicates, and Certified Reference Material (CRM – NIST SRM 1945), listed in Tables S1 and S2. The recovery of the surrogate standard in each sample was calculated based on the ratio between the surrogate peak area and that of the internal standard (IS) and ranged from 71 to 108% for POPs and from 62 to 104% for PAHs, as listed in Table S3. The QA/QC procedures met the established validation criteria, ensuring consistent and reliable analytical performance throughout the study. The method's limit of quantification (LOQ) was established based on the lowest calibration point, set at 3.6 ng g^−1^ wet weight (ww). The limit of detection (LOD) for each compound was calculated based on the variability observed in seven replicate analyses of the same sample. The LOD values for each compound are detailed in Table S4.

## Results and discussion

All contaminant concentrations were expressed on a wet weight basis, as this approach better reflects the actual levels present in the tissue and facilitates comparisons with monitoring datasets and regulatory references. Nevertheless, both moisture and lipid contents were determined and are reported for all samples, allowing the conversion of concentrations to dry weight or lipid weight bases when necessary.

POPs were detected in 100% of the liver samples from Magellanic penguins (*Spheniscus magellanicus*) analyzed in this study. Among the identified contaminants, PCBs showed the highest concentrations, followed by DDTs, HCB, Drins (dieldrin and endrin), mirex and endosulfans (Table 1). The biomagnification of PCBs and DDTs in birds is significantly greater than that of other organochlorine (Kawano et al. 1986; Guruge et al. 1997), likely due to differences in metabolic processing and excretion pathways (Guruge et al. 2001). Despite their persistence, certain organochlorine pesticides, such as hexachlorocyclohexanes (HCHs), heptachlor, and oxychlordane, were not detected in any of the liver samples. Their absence may indicate limited regional use or environmental deposition, suggesting a comparatively lower level of contamination in the study area. Similarly, polybrominated diphenyl ethers (PBDEs), widely used as flame retardants (ATSDR 2017; De Wit 2002), were also undetected, further supporting the hypothesis of reduced environmental exposure to these compounds in the sampled population.

**Table 1:** Persistent organic pollutants content in liver of *S. magellanicus* specimens (ng g^−1^ ww) and lipidic content (%). n = 23; F = female; M = male. Non-available data = n.a

Sample ID	Location	Sex	ΣPCBs	ΣDDTs	HCB	ΣDrins	Mirex	ΣEndosulfans	Lipids (%)
29841	Pântano do Sul	F	4.0	5.8	< LOQ	< LOD	< LOD	< LOD	3.6
30037	Mole	M	49.0	24.8	15.8	4.4	6.7	4.9	0.4
30038	Campeche	M	37.4	21.7	16.7	4.4	5.8	< LOD	2.4
30040	Joaquina	F	44.3	11.8	4.0	< LOD	5.5	< LOD	0.8
31113	Moçambique/Barra da Lagoa	F	< LOD	< LOD	< LOD	< LOD	< LOD	< LOD	2.0
31133	Santinho	M	42.3	22.6	16.2	4.5	4.6	3.6	0.8
31625	Armação	F	94.5	28.3	9.1	6.3	4.5	< LOD	0.4
31769	Joaquina	F	30.5	18.2	30.0	13.6	5.5	7.3	2.4
31788	Morro das Pedras	F	76.8	28.8	< LOD	< LOD	4.5	< LOD	2.4
45839	Moçambique/Barra da Lagoa	F	36.8	29.5	< LOD	5.9	3.6	< LOD	5.2
47473	Moçambique/Barra da Lagoa	F	63.4	20.9	5.1	< LOD	< LOQ	< LOD	4.8
48546	Ingleses	M	116.4	39.1	30.9	18.9	5.2	7.1	2.4
50727	Ingleses	M	72.3	22.5	10.9	3.9	5.3	< LOD	0.4
52615	Matadeiro	F	47.0	13.4	< LOD	< LOD	< LOQ	< LOD	6.0
52903	n.a	F	170.5	47.6	23.5	17.1	13.3	9.6	4.4
53312	Armação	F	43.4	14.3	8.3	5.6	5.2	< LOD	2.0
53674	Joaquina	F	18.2	13.8	7.9	5.2	< LOQ	< LOD	0.4
53705	n.a	F	< LOD	< LOD	< LOD	< LOD	< LOD	< LOD	2.4
54685	Moçambique/Barra da Lagoa	F	31.8	11.0	8.3	< LOD	< LOQ	< LOD	3.2
55328	n.a	F	50.2	26.1	21.1	8.5	5.3	5.4	0.4
55540	Moçambique/Barra da Lagoa	F	199.4	45.3	< LOD	4.9	8.4	< LOD	3.2
55556	Pântano do Sul	F	48.8	22.4	19.1	9.2	< LOQ	3.9	4.4
55633	Santinho	M	39.1	25.6	21.1	11.7	5.7	6.8	0.4

### Polychlorinated biphenyls

PCB concentrations ranged from below the detection limit (0.05 ng g^−1^ ww) to 199.4 ng g^−1^ ww, with an average of 57.2 ± 49.0 ng g^−1^ ww (Table 1). These values are lower than those reported by Baldassin et al. (2016), who found average concentrations of 104 ng g^−1^ ww in liver tissue of *S. magellanicus* from the same region of the Brazilian coast. They are also substantially lower than the concentrations reported by Quinete et al. (2020) for 9 individuals of *S. magellanicus* collected along the coast of Rio de Janeiro, located in southeastern Brazil, where hepatic PCB levels ranged from 228 to 1058 ng g^−1^ ww. These differences highlight both temporal variation and regional differences in contamination levels, which may reflect distinct local sources of pollution, oceanographic conditions, or foraging patterns. In the study by Baldassin et al. (2016), which analyzed 116 individuals from Brazil, Uruguay and Chile, a decreasing trend in PCB concentrations was observed between 2008 and 2012, a pattern that is corroborated by the present findings. As in that study, the individuals analyzed here were also found in poor body condition, ranging from thin to cachectic, and were likely subject to similar migration and ecological conditions. Therefore, among the available environmental variables, a reduction in environmental exposure appears to be the most plausible factor contributing to the observed decrease in PCB concentrations.

Despite this decline, the PCB levels reported here are still up to a thousand times higher than those found in Antarctic penguins such as *Pygoscelis papua* and *P. adeliae*, whose liver concentrations range from 0.1 to 0.9 ng g^−1^ ww (Subramanian et al. 1986; De Boer and Wester 1991; Inomata et al. 1996). However, due to ecological and geographic differences, direct comparisons between these species should be made with caution. While both *S. magellanicus* and *P. papua* consume small fish, crustaceans, and cephalopods, *P. adeliae* has a krill-dominated diet, which may contribute to interspecies variation in PCB accumulation.

**Fig. 2:**
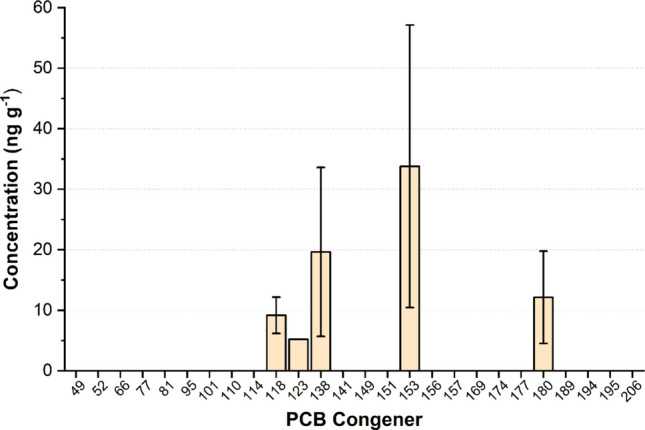
Mean concentrations of PCB congeners among samples. Error bars represent standard deviation (SD)

The most abundant congeners were the hexachlorinated PCBs 153 and 138, accounting for 82% of total PCBs (Fig. 2). PCB 180, a heptachlorinated congener, comprised 14%, while PCB 118, also hexachlorinated, made up the remaining 4%. Although overall concentrations have declined over the years, consistent with the trend reported by Baldassin et al. (2016) and Quinete et al. (2020), the congener profile remains largely unchanged. These patterns reflect the lower metabolic elimination rates of higher chlorinated congeners in birds, particularly those with chlorine atoms in the ortho positions, such as PCBs 153, 138, and 180, which are di-ortho substituted, i.e., bearing two chlorine atoms in ortho positions, and are likely more resistant to biotransformation and excretion, possibly due to the increased torsional angle between the two benzene rings associated with greater ortho substitution (Kannan et al. 1989, 1995). In contrast, PCB 118 is single-ortho substituted, which may influence its slightly different metabolic behavior (Norstrom et al. 1988; Drouillard et al. 2001; Covaci et al. 2002). Thus, selective accumulation appears to be influenced by congener structure, especially chlorine atom position, which affects degradation and excretion efficiency. Additional factors such as sex, age, health, and genetic traits also contribute to individual variability in PCB elimination (Barron et al. 2003).

The ecological relevance of this congener-specific accumulation lies in its potential to cause sublethal effects in seabirds, including penguins. Even at low concentrations, persistent PCBs can interfere with physiological and reproductive functions, compromising the fitness of individuals and potentially affecting population dynamics over time. Documented effects include enzyme induction, suppressed immune function, weight loss, growth impairment, and reproductive disorders. These compounds may also disrupt calcium metabolism, leading to eggshell thinning and reduced hatching success (Yamashita et al. 1993).

### Organochlorine pesticides

Among DDT metabolites, only p,p'-DDE (4,4'-DDE) was detected, ranging from < LOD to 47.6 ng g^−1^ ww (mean: 21.5 ± 12.3 ng g^−1^ ww) (Table 1). This finding is consistent with Baldassin et al. (2016) and Quinete et al. (2020), who also reported p,p'-DDE as the predominant metabolite in liver tissue samples of the same species collected in Florianópolis (< LOD to 129 ng g^−1^ ww) and along the Rio de Janeiro coast (22.9–163 ng g^−1^ ww), respectively. In both studies, other DDT congeners were detected, but only in a few samples and at lower concentrations, further reinforcing the predominance of p,p'-DDE. In contrast to the declining trend observed for PCBs, concentrations of DDT metabolites in individuals sampled in 2017 were similar to those reported by Baldassin et al. (2016) for the period between 2008 and 2012, indicating no significant reduction over time. This persistent presence of p,p'-DDE in seabird tissues reflects its high environmental stability and continued availability in the food chain.

The compound p,p′-DDE is the predominant DDT breakdown product found in avian tissues due to its high persistence (Ohlendorf et al. 1978). The absence of p,p′-DDT in these samples suggests that there are no recent sources or exposure of Magellanic penguins to DDTs. This is consistent with studies showing that p,p′-DDT/p,p′-DDE ratios < 1.0 in several organisms, including Adelie penguin, indicate contamination by older DDT sources rather than recent exposure (Geisz et al. 2008). Indeed, the p,p′-DDT/p,p′-DDE ratio measured in Adelie penguins has significantly declined since 1964 (Geisz et al. 2008), reflecting the predominance of historical rather than current DDT sources in marine food web.

Despite the lack of recent exposure, p,p′-DDE remains a persistent contaminant, and its continued presence in avian tissues has been linked to various sublethal effects. This compound has been associated with eggshell thinning and increased egg breakage, contributing to population declines in several bird species (Wiemeyer et al. 1993; Dirksen et al. 1995). However, the concentrations observed here are three orders of magnitude lower than levels known to cause significant reproductive harm (Lundholm and Bartonek 1992). Additionally, p,p′-DDE may induce hepatic cytochrome P450 enzymes, potentially enhancing the carcinogenicity of co-occurring environmental contaminants by promoting tumor cell proliferation (Wyde et al. 2003).

HCB was detected in 16 out of 23 samples, with concentrations ranging from < LOD to 30.9 ng g^−1^ ww (mean: 10.9 ± 10.0 ng g^−1^ ww) and was probably accumulated in colder regions due global distillation effect (Cipro et al. 2010). These concentrations are similar to those reported by Quinete et al. (2020) (1.62–33.5 ng g^−1^ ww), but lower than the mean value of 35 ng g^−1^ ww reported by Baldassin et al. (2016) for samples collected in the same region, suggesting a possible decline in HCB contamination over time. In Brazil, Baldassin et al. (2012) observed a correlation between HCB exposure and cardiovascular alterations in *Spheniscus magellanicus*, although causality remains unclear (Carvalho et al. 2012). Experimental studies in animal models indicate that HCB can induce neurological symptoms, such as tremors and paresis, even in the absence of histopathological damage (Michielsen et al. 1999). Additionally, iron supplementation, commonly administered during the rehabilitation of anemic penguins, may exacerbate HCB toxicity through lipid peroxidation mechanisms (Alleman et al. 1985), underscoring the need for improved toxicological screening protocols in wildlife rescue centers.

Drins, including dieldrin and endrin, mirex, and endosulfans, were also detected at concentrations comparable to those reported by Baldassin et al. (2016) and Quinete et al. (2020). Dieldrin represented 72% of the total drins, with concentrations ranging from < LOD to 18.9 ng g^−1^ ww (mean: 5.4 ± 5.6 ng g^−1^ ww). Their continued presence is likely associated with historical usage, as aldrin and mirex were not included in ANVISA’s Ordinance No. 329/1985, which permitted their prolonged application in Brazil. Mirex was detected in 65% of samples, with concentrations ranging from < LOD to 13.3 ng g^−1^ ww (mean: 5.4 ± 5.6 ng g^−1^ ww), similar to the levels found by Quinete et al. (2020) (4.25–14.8 ng g^−1^ ww). Endosulfans were detected in 8 out of 23 individuals, with concentrations ranging from < LOD to 9.6 ng g^−1^ ww (mean: 2.1 ± 3.2 ng g^−1^ ww). Environmental incidents, such as the 2008 endosulfan spill in the Pirapitinga River (Rio de Janeiro) (IBAMA 2009; Azevedo-Santos et al. 2025), highlight the persistent nature and ecological risks associated with these compounds.

Although PCBs and organochlorine pesticides were globally banned in the 1970 s and 1980 s, continued monitoring of their environmental patterns and diagnostic ratios, indicating recent or historical exposure remains essential, particularly in the context of glacier melt. Due to their volatility, organochlorines have been transported to temperate, subpolar, and polar regions through global distillation, where they have accumulated in glacial ice. As global temperatures rise, the melting of glaciers may lead to the remobilization of these legacy contaminants, posing renewed risks to ecosystems and wildlife. Thus, glacier melt may act as an emerging secondary source of exposure to these persistent pollutants.

### Polycyclic aromatic hydrocarbons

PAHs were not detected in any of the liver samples analyzed in this study. There are few reports concerning PAHs in penguin tissues, and Quinete et al. (2020) is one of the few studies addressing this issue in *Spheniscus magellanicus*. In that study, most PAHs and related compounds were detected at very low concentrations and were mostly below the limit of quantification. However, a direct comparison between the present study and Quinete et al. (2020) is not feasible due to differences in the analytical targets. Notably, the same authors included compounds such as decalins in their total PAH calculations, despite decalins not being true PAHs, as they lack aromatic rings, as well as other non-aromatic hydrocarbons like C30-hopane. Decalins were among the compounds found in the highest concentrations in their analysis. These methodological differences limit the comparability of PAH concentration data across studies.

The consistent observation of low or undetectable PAH levels in penguin tissues may, in part, be attributed to the efficient metabolic processing and elimination mechanisms characteristic of vertebrates. In particular, PAHs are readily biotransformed in the liver through a series of enzymatic reactions, such as oxidation, reduction, hydrolysis, and conjugation, primarily mediated by cytochrome P450 enzymes within the mixed-function monooxygenase system. These metabolic pathways enhance the hydrophilicity of PAH metabolites, facilitating their excretion through biological fluids (Klaassen et al. 1996). This detoxification mechanism, which includes the formation of reactive intermediates like diol-epoxides, is among the most well-characterized for PAH bioactivation and clearance (Hall et al. 1989; Akcha et al. 2000). Therefore, the absence of detectable PAHs in the present study likely reflects both analytical limitations and the biological capacity of penguins to efficiently metabolize and eliminate these compounds, even under conditions of potential environmental exposure (Hylland, 2006).

### Toxicity of PCBs

To understand and contextualize the toxicity potential of the PCB results, the toxic equivalents (TEQ) were calculated, utilizing the toxicity equivalent factors in birds proposed by the World Health Organization (WHO) (Van Den Berg et al. 1998). Of the 12 PCBs with TEF values established by WHO, only 2 were detected in this study (PCB 118 and 123). Calculated TEQ values ranged from 0.05 to 0.14 pg g^−1^ ww, and were considered particularly low, especially considering the 210 pg g^−1^ threshold which has been reported as the generic limit for harmful effects in birds (Elliott et al. 1996). TEQ values were similar to those reported by Motas et al. (2025a, b), who evaluated PCBs in liver, muscle and brain tissue of chinstrap penguins from Antarctica, and found mean values of 0.167 pg g^−1^ ww. Our results were considerably lower than those reported by Quinete et al. (2020), who also evaluated PCBs in magellanic penguins from the southeastern coast of Brazil and found TEQ values of 0.16–8.5 pg g^−1^ ww. Morales et al. (2022) also found higher TEQ concentrations in gentoo and chinstrap penguins from Antarctica, ranging from 1.38–7.33 pg g^−1^ lw. Nevertheless, considering only mono-ortho PCBs, which were the ones detected in our study, the TEQ values were similar, ranging from 0.002–0.06 pg g^−1^ lw and 0.01–0.09 pg g^−1^ lw, in chinstrap and gentoo penguins, respectively.

## Conclusion

This study confirms the widespread presence of persistent organic pollutants (POPs) in the liver tissues of Magellanic penguins (*Spheniscus magellanicus*) along the coast of Santa Catarina, Brazil. The detection of PCBs, DDTs, HCB, Drins, mirex, and endosulfans in juveniles highlights the bioaccumulative nature of these substances and their ongoing circulation in marine ecosystems, despite historical bans. Among the POPs analyzed, PCBs exhibited the highest concentrations, with congener profiles consistent with previous studies, reflecting both selective retention mechanisms and persistent environmental presence.

Although a decreasing trend in some contaminant levels, particularly PCBs and HCB, was observed compared to earlier reports, the persistent detection of p,p'-DDE and other legacy pesticides underscores the long-term ecological risks posed by these compounds. Even at sublethal concentrations, their presence may contribute to physiological stress, impaired health, and reduced fitness in seabirds, particularly during energetically demanding life stages such as migration and early development.

In contrast, PAHs were not detected in any of the liver samples analyzed, likely due to their efficient metabolic processing and rapid elimination in penguins. These compounds are readily biotransformed in the liver through enzymatic pathways mediated by cytochrome P450 enzymes, which enhance their solubility and excretion, resulting in low or undetectable tissue concentrations.

Given the role of seabirds as sentinel species for marine pollution, ongoing monitoring of POPs in Magellanic penguins is crucial for assessing environmental health and identifying emerging threats. Additionally, global climate change and glacial melting may reintroduce previously sequestered contaminants into the environment, further emphasizing the need for proactive toxicological surveillance and mitigation strategies. Understanding contaminant dynamics in sentinel species like *S. magellanicus* not only informs conservation efforts but also enhances our broader understanding of pollutant behavior in the Southern Hemisphere.

## Supplementary Information

Below is the link to the electronic supplementary material.

Supplementary file1 (DOCX 62 KB)

## Data Availability

All data generated or analyzed during this study are included in this published article.
